# *Cis*-Acting Pathways Selectively Enforce the Non-Immunogenicity of Shed Placental Antigen for Maternal CD8 T Cells

**DOI:** 10.1371/journal.pone.0084064

**Published:** 2013-12-31

**Authors:** Chin-Siean Tay, Elisa Tagliani, Mary K. Collins, Adrian Erlebacher

**Affiliations:** 1 Department of Pathology, NYU School of Medicine, New York, New York, United States of America; 2 NYU Cancer Institute, NYU School of Medicine, New York, New York, United States of America; Otto-von-Guericke University Magdeburg, Germany

## Abstract

Maternal immune tolerance towards the fetus and placenta is thought to be established in part by pathways that attenuate T cell priming to antigens released from the placenta into maternal blood. These pathways remain largely undefined and their existence, at face value, seems incompatible with a mother's need to maintain a functional immune system during pregnancy. A particular conundrum is evident if we consider that maternal antigen presenting cells, activated in order to prime T cells to pathogen-derived antigens, would also have the capacity to prime T cells to co-ingested placental antigens. Here, we address this paradox using a transgenic system in which placental membranes are tagged with a strong surrogate antigen (ovalbumin). We find that although a remarkably large quantity of acellular ovalbumin-containing placental material is released into maternal blood, splenic CD8 T cells in pregnant mice bearing unmanipulated T cell repertoires are not primed to ovalbumin even if the mice are intravenously injected with adjuvants. This failure was largely independent of regulatory T cells, and instead was linked to the intrinsic characteristics of the released material that rendered it selectively non-immunogenic, potentially by sequestering it from CD8α^+^ dendritic cells. The release of ovalbumin-containing placental material into maternal blood thus had no discernable impact on CD8 T cell priming to soluble ovalbumin injected intravenously during pregnancy, nor did it induce long-term tolerance to ovalbumin. Together, these results outline a major pathway governing the maternal immune response to the placenta, and suggest how tolerance to placental antigens can be maintained systemically without being detrimental to host defense.

## Introduction

A key feature of pregnancy is the failure of the maternal immune system to mount immunogenic T cell responses to the placenta. Recent work has suggested that this failure is in part due to limitations in available antigen presentation pathways. In contrast to surgical organ transplants, which are rejected primarily by host T cells that directly engage MHC molecules on donor cells, the placenta is thought to be recognized by maternal T cells that predominantly, if not exclusively, engage minor histocompatibility and tissue-specific antigens presented by maternal antigen presenting cells (APCs) (for review, see [1], [2]). These insights have come from experimental systems that involve transgenic expression of well-characterized model antigens in mice. For example, in the Act-mOVA system, Act-mOVA transgenic males are crossed with non-transgenic females to generate concepti that express a transmembrane form of chicken egg ovalbumin (mOVA) as a surrogate placental antigen [3], [4]. Maternal T cell responses to this antigen are then monitored through the use of adoptively transferred ovalbumin- (OVA-) specific TCR transgenic T cells, namely OT-I CD8 T cells and OT-II CD4 T cells. In this system, mOVA starts being shed into maternal blood at around embryonic day (E) 10.5 [3].

Despite robustly proliferating throughout all secondary lymphoid organs, CD8 and CD4 T cells specific for placental antigen fail to expand substantially in number or differentiate into effector cells [3]–[5]. Within the uterine lymph nodes (LN), this observation can in part be explained by the inability of uterine-resident dendritic cells (DCs) to exit the maternal-fetal interface [6]. Systemically, the non-immunogenic nature of placental antigen presentation has largely been attributed to the activity of regulatory T cells (Treg cells) (for review, see [7], [8]). This idea has come from the observation that the acute depletion of Treg cells at mid-gestation increases the expansion of adoptively transferred OT-I and OT-II cells in the spleens of Act-mOVA-mated pregnant mice, and increases the effector function of the transferred OT-I cells [9]. Treg cell-mediated immunosuppression might also explain prior results that intravenous adjuvant injection into pregnant females bearing both Act-mOVA^+^ concepti and large numbers of OT-I T cells induces submaximal levels of OVA-specific cytotoxic T lymphocyte (CTL) activity [3]. Treg cell numbers have also been noted to expand during pregnancy, in particular those that recognize certain placental antigens, and depletion of Treg cells has been shown to induce fetal loss [5], [9]–[12].

However, certain conceptual difficulties arise from the idea that pregnancy success critically relies upon the inhibition of placenta-specific T cells by Treg cells. If Treg cells act in pregnant animals in part as they do in non-pregnant animals, i.e. by inhibiting the generation of effector T cells *in trans* via antigen non-specific mechanisms (bystander suppression), it is difficult to understand why the high levels of immunosuppression presumably required to prevent T cell activation to the set of all placental antigens does not significantly compromise host defense. Indeed, infection susceptibility during gestation is generally increased only with microbes that colonize the maternal-fetal interface (for review, see [13]). Conversely, the ability of inflammation to countermand Treg cell function [14] raises the possibility that non-uterine infections or sterile tissue damage might induce T cell priming to placental antigens as a collateral effect. Treg cell inhibition leading to anti-fetal/placental T cell priming has in fact been proposed to help explain why systemic *Listeria monocytogenes* infection in mice induces fetal resorption [15], but the exact causal relationships between infection-induced inflammation, Treg cell inhibition, T cell priming, and fetal loss in this model have not been definitively established [1].

Together, these considerations underscore that our understanding of the pathways that modulate maternal T cell responses to fetal and placental antigens is still quite rudimentary. Indeed, the current literature is also incomplete in that all work to date on the CD8 T cell component of these responses has relied upon the adoptive transfer of relatively high numbers of TCR transgenic T cells, which can generate non-physiological results [16]. Here, we again use the Act-mOVA transgenic system to study how maternal CD8 T cells respond to shed placental antigen, but now base our analysis on the behavior of endogenous CD8 T cells. Our results reveal that the maternal CD8 T cell response to shed placental antigen is governed by a novel, Treg cell-independent pathway that renders this antigen selectively and profoundly non-immunogenic without compromising CD8 T cell responses to exogenous antigen.

## Materials and Methods

### Ethics statement

All work with mice was conducted according to relevant national and international guidelines. All procedures were approved by the Institutional Animal Care and Use Committee (IACUC) of NYU Langone Medical Center.

### Mice and reagents

Hemizygous Act-mOVA [17] and C57BL/6 (B6) mice were from The Jackson Laboratory and Taconic Farms, respectively. *Ccr7^-/-^* mice [18] on a B6 background were the gift of Sergio Lira (Mt. Sinai School of Medicine). OT-I and OT-II mice were from Taconic Farms; OT-I CD45.1 mice were generated as previously described [19]. Mice were housed in a specific pathogen-free facility. Noon of the day of the copulation plug was E0.5. Genotyping was performed as described previously [3].

OVA was purchased from Sigma-Aldrich (fraction VI) and was rendered endotoxin-free through the use of Detoxi-Gel columns (Pierce). Anti-CD40 (clone FGK45) and anti-CD25 (clone PC61) Abs were purchased from Bio-X-Cell. Poly(I:C) was from Invivogen. K^b^/OVAp tetramers were produced by the NYU Cancer Institute's Immune Monitoring core facility. OVAp was from Anaspec, Inc. Fluorochrome-conjugated Abs to CD8α (clone 53-6.7), CD44 (IM7), CD45.1 (A20), CD11c (N418), MHCII (M5/114.15.2), B220 (RA3-6B2), CD4 (RM4-5), CD11b (M1/70), CD86 (GL-1), CD25 (7D4), FOXP3 (FJK-16S), and IFNγ (XMG1.2) were from Biolegend and eBioscience.

### Biochemical assessments of mOVA in maternal plasma

Mice were injected intravenously with 5 USP units heparin 5 min before sacrifice and blood collection. Blood was centrifuged at 300×*g* to remove cells, and then sequentially centrifuged as indicated. 50 µl of the indicated plasma fraction, or the corresponding amount of pelleted material, was then incubated with rabbit anti-OVA Abs (Fitzgerald Industries International) followed by dynabead-coupled sheep anti-rabbit Ig (Invitrogen) overnight at 4°C in a total volume of 0.8–1.0 ml. Half of the immunoprecipitated material was then subjected to non-reducing western blot analysis using mouse anti-OVA MoAbs (clone 1E7, Abcam).

### Treatments

Cells and reagents were administered via retro-orbital injection. Mice were sacrificed 6 days after adjuvant injection. In mice given both adjuvants and sOVA, these reagents were given at the same time. OT-I and OT-II cells were prepared using MACS columns (Miltenyi) and labeled with CFSE (Invitrogen) as previously described [3]. OT-I (1×10^6^ cells/mouse) and OT-II (2×10^6^ cells/mouse) CFSE dilution profiles were determined 44 and 60 h after injection, respectively. PC61 Abs (500 µg) were injected one day prior to adjuvant±sOVA injection. The effect of anti-CD40 Abs and poly(I:C) on DC CD86 expression was determined in virgin B6 or E16.5-18.5 pregnant (B6 X B6) mice 24 h after injection.

### Flow cytometry and intracellular staining

These analyses were performed as previously described [3]. Intracellular IFNγ staining was performed after stimulating the cells with 10 µM OVAp for 6 hours, with brefeldin added for the last 2 h. Intracellular staining with anti-FOXP3 Abs was performed according to the manufacturer's instructions (eBioscience). The percentage of CD86^+^ cells in splenic DC subsets was determined using an arbitrarily set marker. CD8α^+^ DCs were identified as cells with a CD11c^hi^ B220^−^ CD8α^+^ CD11b^lo^ surface phenotype; CD4^+^ DCs as cells with a CD11c^hi^ B220^−^ CD8α^−^ CD11b^+^ CD4^+^ phenotype; CD8α^−^ CD4^−^ DCs as a cells with a CD11c^hi^ B220^−^ CD8α^−^ CD11b^+^ CD4^−^ phenotype.

### Statistical analysis

All comparisons were performed using a two-tailed Student's *t*-test, with a *P* value <0.05 considered to be statistically significant.

## Results

### The mouse placenta releases large amounts of antigen into maternal blood during late gestation

**Figure 1:**
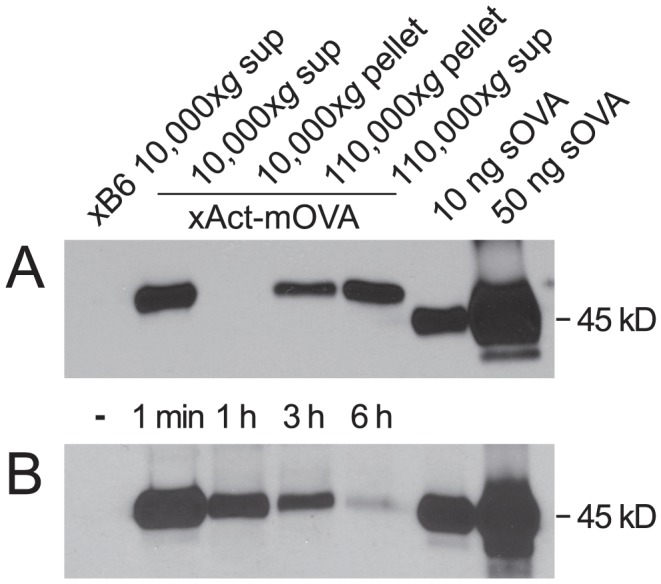
Shedding of mOVA into maternal blood. OVA immunoprecipitation followed by anti-OVA western blotting was performed on plasma samples. For comparison, 10 and 50 ng of sOVA were directly loaded into the last two lanes of each gel. (A) Plasma from B6- and Act-mOVA-mated pregnant mice was pooled (*n* = 4 mice per pool) and purified by differential centrifugation. The amount of material loaded into each lane was derived from the same initial amount of plasma (25 µl). (B) sOVA clearance from mouse plasma. Mice were uninjected (-) or injected with 100 µg sOVA. Plasma was obtained at the indicated times post-injection. Both blots are representative of two independent experiments.

Although the systemic proliferative response of transferred OVA-specific OT-I T cells readily demonstrates mOVA release from Act-mOVA^+^ concepti into maternal blood [3], the amount of this antigen and its physical form have remained unclear. Therefore, we employed biochemical methodologies to visualize mOVA release from the placenta. Remarkably, anti-OVA immunoprecipitation and western blotting revealed that the plasma of late gestation (E15.5-16.5) females impregnated by Act-mOVA males contained, in pooled samples, about 400 ng/ml anti-OVA-immunoreactive protein (Fig. 1A). This protein ran at a higher apparent molecular weight than soluble OVA (sOVA), consistent with the mOVA construct's transmembrane domain. Shed mOVA thus likely remains membrane-associated, as we had inferred from earlier work [3], [19]. mOVA was not pelleted when maternal plasma was centrifuged at 10,000×*g* (Fig. 1A), indicating that it was not associated with large cellular debris or apoptotic microparticles, but ∼1/3^rd^ of it was pelleted when the 10,000×*g* supernatant was ultracentrifuged at 110,000×*g*, a force that pellets exosomes. A portion of the mOVA might therefore be exosome-associated, in accord with the abundant release of this microvesicular species from the human placenta [20]. We speculate that the unpelletable material is comprised of placenta-derived membrane fragments.

Consistent with prior results [21], intravenously injected sOVA was rapidly cleared from the plasma, with a half-life on the order of 1 hour (Fig. 1B). Moreover, the amount of sOVA remaining in the plasma after 1 h was on par with the amount of mOVA present, on average, at steady state in late gestation pregnant females. These data thus suggest a remarkably high level of constitutive mOVA release from the conceptus, potentially on the order of hundreds of micrograms per day. Although we cannot rule out the possibility that some of the mOVA is derived from the fetus proper and reaches the maternal circulation via transplacental transport, it is likely that the vast majority of it is placenta-derived and will be referred to hereafter as such.

### Placental mOVA is non-immunogenic for CD8 T cells, even in mice injected intravenously with adjuvants

**Figure 2:**
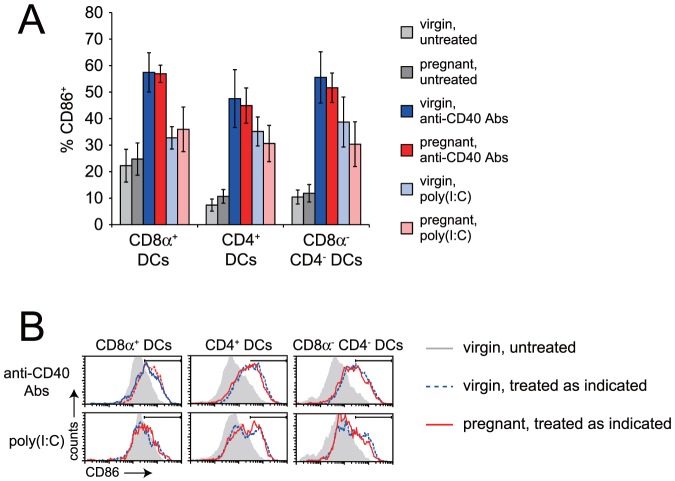
Pregnancy does not induce DC activation in the spleen, nor does it inhibit adjuvant-induced DC activation. (A) Induction of CD86 expression by splenic DC subsets in virgin and pregnant mice by anti-CD40 Abs or poly(I:C). Data show *n* = 4–12 mice per group (mean±SD), from at least 2 independent experiments per group. *, *P*<0.001. (B) Representative histograms. For clarity, untreated pregnant mice are not shown.

Next, we assessed the circumstances under which shed placental mOVA could prime endogenous CD8 T cells. To this end, we monitored the expansion of OVA-specific CD8 T cells in the spleen using tetramers comprised of H-2K^b^ (K^b^) bound to the immunodominant OVA peptide OVA_257–264_ (OVAp), the same MHC/peptide complex recognized by OT-I TCR transgenic T cells. Since splenic DCs do not spontaneously activate during pregnancy (Fig. 2A), we anticipated that the responding T cells might fail to expand simply because of a lack of costimulation. Therefore, some mice were injected intravenously with agonistic anti-CD40 antibodies (Abs) and polyinosinic:polycytidylic acid (poly(I:C)), an adjuvant combination that stimulates robust CD8 T cell priming [22]. The use of anti-CD40 Abs also had the advantage of mimicking CD4 T cell help, and thus circumventing any potential tolerization of the CD4 T cell compartment. Anti-CD40 Abs and poly(I:C) were given at doses of 100 µg and 15 µg, respectively, which induced equivalent levels of CD86 upregulation by splenic DCs in pregnant and virgin mice (Fig. 2). Anti-CD40 Abs+poly(I:C) injection at midgestation had no detectable effect on litter size in Act-mOVA-impregnated females (7.0±2.1 pups in untreated mice versus 7.1±2.2 pups in adjuvant-injected mice [mean±SD]; *n* = 8 and 7 mice per group, respectively), thus allowing us to avoid the potentially confounding effects of embryo resorption on the magnitude and quality of the anti-fetal/placental T cell response.

**Figure 3:**
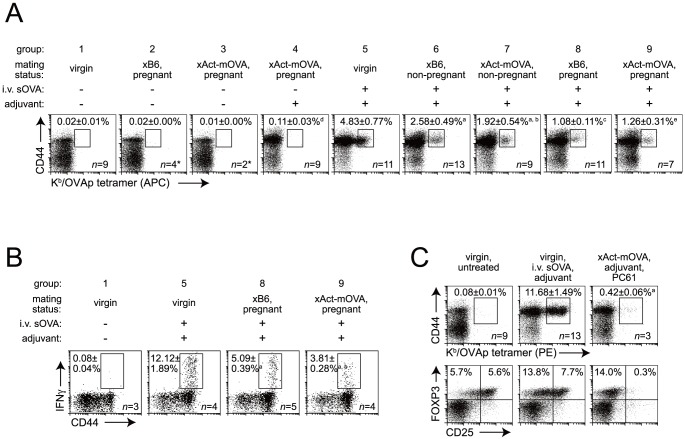
Non-immunogenic responses of maternal CD8 T cells to shed placental mOVA. (A) Effects of mating, pregnancy, adjuvant (anti-CD40 Abs +poly(I:C)), sOVA, and mOVA on the expansion of OVA-specific CD8 T cells. Adjuvant±sOVA was injected 6 days prior to sacrifice. Representative dot plots and mean±SEM of the percentage of CD44^hi^ APC-conjugated K^b^/OVAp-tetramer^+^ cells of total splenic CD8 T cells. Pregnant mice were killed on E17.5 to 1 day after delivery. Mated mice that failed to become pregnant were killed on what would have been E17.5-21.5. Aside from group 3 (one experiment), data are from at least 4 independent experiments per group. In addition (*), *n* = 7 group 2 and *n* = 4 group 3 mice showed no difference compared to *n* = 9 virgin, untreated (group 1) mice when analyzed with PE-conjugated K^b^/OVAp-tetramers (2-8 experiments). a, *P*<0.02 vs. group 4; b, *P* = 0.38 vs. group 5; c, *P* = 0.01 vs. group 5; d, *P* = 0.01 vs. group 1; e, *P* = 0.53 vs. group 7. (B) Effects of pregnancy and mOVA shedding from the placenta on the induction of IFNγ-expressing splenic CD8 T cells by intravenous sOVA+adjuvant injection. Experimental groups are a subset of those shown in panel A. Representative dot plots and mean±SEM of the percentage of CD44^hi^ IFNγ+ cells of total splenic CD8 T cells. Splenocytes were treated *ex vivo* with OVAp prior to analysis. Data are from two independent experiments. a, *P* = 0.005 vs. group 5; b, *P* = 0.04 vs. group 8. (C) Effect of PC61-mediated Treg depletion on OVA-specific CD8 T cell expansion in Act-mOVA-mated pregnant mice treated with anti-CD40 Abs+poly(I:C). Top row: representative dot plots and mean±SEM of the percentage of CD44^hi^ PE-conjugated K^b^/OVAp-tetramer^+^ cells of total splenic CD8 T cells. a, *P*<0.001 vs. virgin, untreated. Of note, due to their increased brightness, the PE-conjugated K^b^/OVAp tetramers used in this experiment detected about twice as many cells as the APC-conjugated K^b^/OVAp tetramers used in the experiment shown in Fig. 3A. Bottom row: representative plots showing loss of FOXP3^+^ CD25^hi^ cells at the time of sacrifice. Data are from 2–8 independent experiments.

As anticipated from previous studies with transferred OT-I cells [3], endogenous OVA-specific CD8 T cells did not detectably expand during late gestation in B6 females impregnated by Act-mOVA males. These mice instead showed the same background level of CD44^hi^ K^b^/OVAp-tetramer^+^ events seen in untreated virgin females and untreated B6-mated pregnant females (Fig. 3A, groups 1–3). Unexpectedly, however, the single combined injection of anti-CD40 Abs+poly(I:C) (i.e. ‘adjuvant’) on E11.5-13.5, six days before sacrifice, induced only a minimal increase in the percentage of CD44^hi^ K^b^/OVAp-tetramer^+^ CD8 T cells (group 4). In contrast, robust OVA-specific CD8 T cell expansion was induced in virgin females injected with adjuvant+sOVA, with the sOVA given at a dose of 100 µg to approximate the lower bound of the amount of mOVA likely shed from the placenta (group 5). Thus, placental mOVA was non-immunogenic even under conditions of strong, adjuvant-induced DC activation.

To explain this result, we first determined how copulation and pregnancy *per se* affected CD8 T cell priming. As compared to the ∼5% seen in virgins (Fig. 3A, group 5), the percentage of CD44^hi^ K^b^/OVAp-tetramer^+^ CD8 T cells induced by sOVA+adjuvant injection was significantly reduced to ∼2.5% in females that had copulated with B6 males but failed to become pregnant (group 6), and was further reduced to ∼1.0% in pregnant, B6-mated females (group 8). Moreover, females that had copulated but failed to become pregnant showed the same level of CD8 T cell expansion irrespective of mating partner (B6 or Act-mOVA; groups 6 and 7), indicating that antigen-specific T cell responses to mOVA in semen, although well documented [4], did not induce antigen-specific tolerance. Together, these data revealed two superimposed tiers of antigen non-specific immunosuppression induced systemically during pregnancy – one that results from copulation *per se* and one that follows successful implantation.

### *Cis*-acting pathways enforce the non-immunogenicity of shed placental mOVA

We consider Treg cells to be likely contributors to these two tiers of antigen non-specific immunosuppression. Treg cells locally expand in the uterine LN following copulation [23], systemically expand by mid-gestation [9], [10], and can limit the magnitude of immunogenic CD8 T cell responses in non-pregnant mice by 2-3-fold [24]–[26]. Furthermore, the ∼3-fold expansion of adoptively transferred OT-I T cells that occurs in response to shed mOVA [3] can be increased ∼5-fold further by transient rectification of the pregnancy-associated increase in Treg cell numbers [9]. However, it seemed unlikely that such quantitative effects could explain the minimal expansion of CD8 T cells in adjuvant-injected Act-mOVA-mated pregnant mice, especially since CD40 ligation is an inflammatory stimulus known to override Treg cell function [27].

To pursue this line of reasoning further, we asked whether the shedding of placental mOVA interfered with the CD8 T cell priming to exogenous sOVA, administered intravenously. Strikingly, we found that the single combined injection of sOVA+adjuvant on E11.5-15.5 induced the same degree of OVA-specific CD8 T cell expansion in B6- and Act-mOVA-mated pregnant mice (Fig. 3A, compare groups 8 and 9). Moreover, even though the proportion of IFNγ^+^ CD8 T cells in *ex vivo* OVAp-stimulated splenocytes from immunized B6-mated pregnant mice was reduced ∼2.4-fold compared to immunized virgins (Fig. 3B, compare groups 5 and 8), consistent with their reduced proportion of K^b^/OVAp-tetramer^+^ CD8 T cells (Fig. 3A), this proportion was reduced only slightly further in OVAp-stimulated splenocytes from Act-mOVA-mated mice (*P* = 0.04; Fig. 3B, compare groups 8 and 9). Thus, the non-immunogenic nature of mOVA presentation to CD8 T cells did not preclude concurrent CD8 T cell priming to intravenously injected sOVA. By logical extension, this result also revealed that impaired CD8 T cell priming to shed mOVA was largely independent of dominant, *trans*-acting immunosuppression, as induced for example by Treg cells or a reduction in IL-2 bioavailability (which is thought to mediate the influence of Treg cells over CD8 T cells [25], [26]). Consistent with this interpretation, robust OVA-specific CD8 T cell expansion also did not occur in adjuvant-injected Act-mOVA-mated pregnant females given anti-CD25 Abs (clone PC61) (Fig. 3C). These Abs deplete FOXP3^+^ CD25^+^ cells, which are thought to comprise the fraction of total FOXP3^+^ Treg cells that contain both inducible Treg cells and the Treg cells that mediate the IL-2-dependent effects over CD8 T cells [26], [28], without impairing CD8 T cell priming [26].

**Figure 4:**
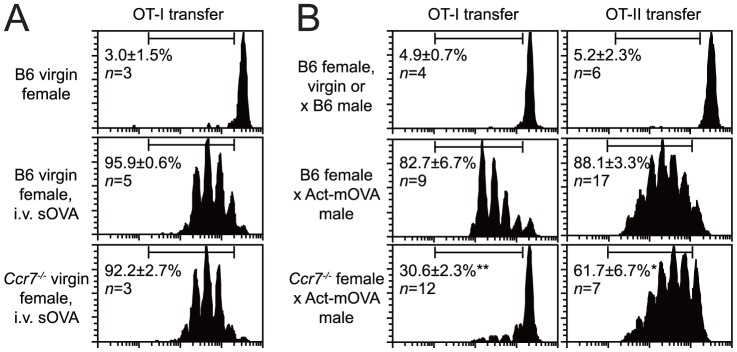
Selectively impaired mOVA presentation to CD8 T cells in *Ccr7^-/-^* mice. Representative CFSE dilution profiles and the percentage of cells having undergone at least one division cycle for all mice in the group (mean±SEM). All data are from 2–5 independent experiments. (A) Response of OT-I T cells transferred into virgin females injected with 100 µg sOVA on the day of T cell transfer. (B) Response of OT-I and OT-II T cells transferred into virgin or E12.5-17.5 pregnant females mated with the indicated partners. * *P* = 0.001, ** *P*<0.0001 compared to corresponding Act-mOVA-mated B6 controls.

Taken together, these data suggested that *cis*-acting pathways induced by factors physically associated with the mOVA-containing placental material were critical for rendering mOVA non-immunogenic. These pathways either might render any APC ingesting mOVA unable to prime CD8 T cells, or channel mOVA for uptake by a distinct APC subset that is either intrinsically non-immunogenic or rendered non-immunogenic by additional factors associated with the shed material. In support of the idea of antigen channeling, we found that mice deficient in CCR7, a chemokine receptor that regulates intra-splenic leukocyte trafficking [18], showed much less proliferation of adoptively transferred OT-I cells as compared to wild-type when impregnated by Act-mOVA males (Fig. 4B, left column), but no change in proliferation when injected with 100 µg sOVA (Fig. 4A). Thus, mOVA and sOVA were being presented by distinct APC subsets. Furthermore, CCR7 deficiency only modestly affected the proliferation of transferred OVA-specific OT-II TCR transgenic CD4 T cells (Fig. 4C, right column), which, given the lower intrinsic responsiveness of OT-II cells as compared to OT-I cells [29], indicated that distinct APC subsets also presented mOVA to CD8 T cells versus CD4 T cells.

### A novel pathway that prevents immune activation without inducing immune tolerance

**Figure 5:**
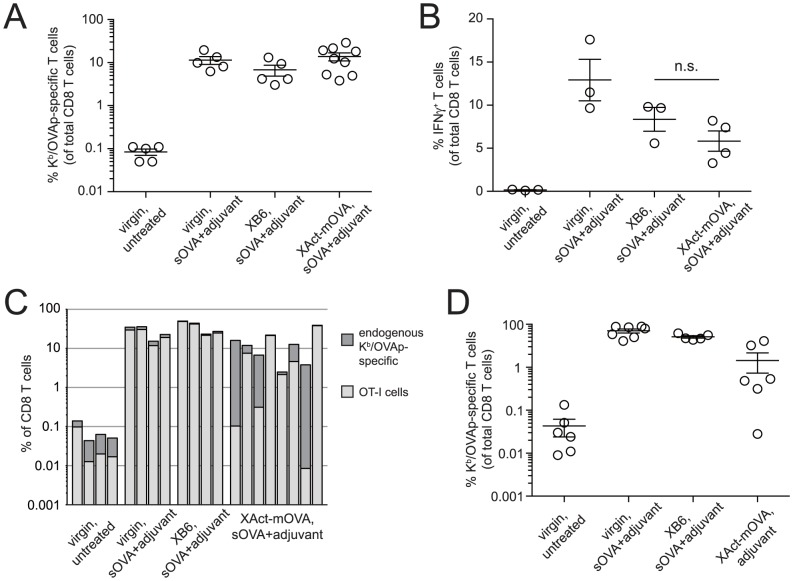
Incomplete deletion and a lack of tolerance induction in maternal CD8 T cells exposed to mOVA. (A) OVA-specific CD8 T cell expansion in postpartum Act-mOVA-mated females. Mice were challenged with sOVA+adjuvants either as virgins or 7–10 days postpartum following pregnancies sired as indicated. Six days later, the percentage of CD44^hi^ PE-conjugated K^b^/OVAp-tetramer^+^ cells of total splenic CD8 T cells was determined. (B) Splenocytes from subsets of the same mice were treated *ex vivo* with OVAp and then stained intracellularly with IFNγ Abs. n.s., not significant. (C) Mice were given 10,000 CD45.1 OT-I T cells on E5.5-8.5, then challenged as in (A) 8–9 days postpartum. Groups of virgin mice were treated and analyzed in parallel. The OT-I cells were identified as being CD45.1^+^; endogenous OVAp-specific cells were identified with PE-conjugated K^b^/OVAp-tetramers. All XAct-mOVA postpartum females analyzed are shown; all other groups show four representative mice from *n* = 4–5 mice per group. (D) Mice were given 5×10^5^ CD45.1 OT-I T cells either as virgins or on E12.5-13.5. Some mice were then injected on the same day with sOVA+adjuvants, or with adjuvants alone. The percentage of OT-I cells was determined 6 days later. All data are from at least 2 independent experiments.

TCR signaling in the absence of sufficient costimulation induces a stereotypical program of CD8 T cell proliferation followed by clonal deletion, with the cells that remain alive less capable of responding to subsequent antigenic challenge (anergy). Surprisingly, however, female mice challenged with sOVA plus adjuvant (anti-CD40 Abs+poly(I:C)) ∼1 week after Act-mOVA^+^ pup delivery showed robust OVA-specific CD8 T cell expansion, with percentages of CD44^hi^ K^b^/OVAp-tetramer^+^ cells similar to those seen in both virgin mice and postpartum females impregnated by B6 males (Fig. 5A). Moreover, OVAp stimulation of *ex vivo* cultured splenocytes from these mice induced the same level of IFNγ expression by CD8 T cells compared to B6-mated postpartum mice, although these levels both appeared mildly reduced compared to virgin controls (Fig. 5B). Thus, aside from a mild, antigen non-specific effect that we suspect is related to the two tiers of immunosuppression documented above (Fig. 3A, B and related discussion), exposure to mOVA during pregnancy did not induce CD8 T cell tolerance to OVA.

One explanation for this result is that mOVA-induced T cell deletion was inefficient and left behind cells fully capable of responding to antigen in the immediate postpartum period. Even if the number of these cells differed dramatically between mice, recent evidence suggests that immunization would induce their expansion to the same numerical “ceiling” [16], [30]. To explore this possibility further, we assessed how well exogenously administered OT-I T cells persisted through Act-mOVA-sired pregnancies. We transferred 10,000 cells, which, given the estimated 10–30% “take” of transferred TCR-transgenic T cells [16], was expected to give rise to only about 8–23 fold more cells than the ∼130 endogenous K^b^/OVAp-specific naïve CD8 T cells thought to reside within B6 mice [31]. The cells were transferred on E5.5-8.5, which is subsequent to the wave of mOVA presentation in the uterine LN induced by insemination [4], but prior to the onset of placental mOVA shedding into maternal blood [3]. As shown in Fig. 5C, the transferred OT-I cells dominated the response in virgin and B6-mated females to sOVA+adjuvant challenge as they always comprised over 75% of all the expanded cells. In contrast, OT-I cells transferred into Act-mOVA-mated females comprised between 0.2% and 99% of the expanded cells. This pattern suggested that mOVA induced a variable level of CD8 T cell deletion, with at least some of the surviving cells remaining competent for activation. It is also possible that a component of the response reflects the thymic production of new OVA-specific cells in the immediate postpartum period.

High numbers of OT-I cells (5×10^5^) also showed a variable response to mOVA during pregnancy itself, with adjuvant injection inducing robust expansion in only some Act-mOVA-mated mice (Fig. 5D). In contrast, sOVA+adjuvant injection induced uniform robust expansion in virgins and B6-mated pregnant females. In the two Act-mOVA-mated mice with the greatest expansion, 32% and 40% of the cells produced IFNγ, indicating priming. The response did not correlate to the number of Act-mOVA^+^ pups in the litter (which in turn did not correlate with plasma mOVA levels; data not shown), and its variability was consistent with our previous finding that adjuvant injection only occasionally induced high OVA-specific CTL activity in Act-mOVA-mated females receiving 1×10^6^ OT-I T cells [3]. Thus, high precursor frequency sometimes allowed the cells to escape the strictures imposed by mOVA-presenting APCs.

## Discussion

In the present study, we analyzed the maternal CD8 T cell response to OVA expressed in transmembrane form as a surrogate placental antigen and shed into the maternal circulation. We found that the systemic presentation of this antigen was highly non-immunogenic, in that it failed to induce the expansion of OVA-specific CD8 T cells not only under steady state, non-inflammatory conditions, but also when pregnant mice were intravenously injected with the strong adjuvant combination of anti-CD40 Abs and poly(I:C). On the other hand, pregnant mice bearing Act-mOVA^+^ concepti were not permanently tolerized to OVA and could be immunized during pregnancy to OVA injected intravenously in soluble form. To our knowledge, the features of this response are quite distinct from the typical features of CD8 T cell peripheral tolerance induction [32], and suggest that *cis*-acting pathways induced by factors physically associated with shed placental mOVA, rather than *trans*-acting immunosuppression, are of primary importance for minimizing its immunogenicity for maternal CD8 T cells.

Importantly, our inability to induce an immunogenic CD8 T cell response to placental mOVA cannot be explained by a paucity of antigen. To the contrary, we found that mOVA was present at about 400 ng/ml plasma at steady state in late gestation, suggesting a remarkably high level of constitutive shedding into the maternal circulation. Indeed, given the ∼1 h half-life of sOVA in mouse blood, we estimate that on the order of hundreds of micrograms of mOVA might be released daily from the placenta during late gestation. This level is consistent with the gram quantities of material released daily from the third trimester human placenta [33]. Furthermore, we found that shed placental OVA retained its transmembrane domain, consistent with it remaining membrane associated. We thus presume that the *cis*-acting pathways that enforce the non-immunogenicity of mOVA will similarly enforce the non-immunogenicity of other proteins associated with shed placental membranes. Since the release of microvesicles and other membrane-associated material is thought to be a major pathway that disseminates placental antigens throughout the mother's body [20], [33], [34], the immune modulatory pathway we have uncovered here may thus be relevant to a great deal of the overall potential CD8 T cell response towards the placenta. Direct evaluation of these points, however, will require the identification of endogenous antigens released from the mouse placenta, which are currently unknown.

Of note, the idea that *cis*- rather than *trans*-acting immunosuppression primarily limits CD8 T cell priming to placental mOVA argues against a major role for Treg cells as mediators of the immunosuppression. This argument is further supported by the fact that one of the adjuvants we employed to activate maternal APCs (anti-CD40 Abs) has previously been shown to countermand Treg cell function [27], as well as by our finding that CD8 T cell priming to shed mOVA was not observed in adjuvant-treated pregnant mice depleted of CD25^+^ Treg cells. On the other hand, given that Treg cells expand following copulation and during pregnancy [9], [10], [23], we suspect that these cells contribute to the moderate degree of antigen non-specific immunosuppression that was evident in pregnant mice. Specifically, following the injection of sOVA and adjuvants, we detected a ∼2-fold reduction in the fold-expansion of CD8 T cells in mice that had copulated with B6 males ∼14 days earlier but failed to become pregnant, and another ∼2.5-fold reduction in B6-mated mice that became pregnant. These effects are relatively minor compared to the virtual complete lack of CD8 T cell priming seen with shed mOVA, but are consistent with the documented degree to which Treg cells limit the magnitude of CD8 T cell expansion [24]–[26]. Importantly, we cannot entirely exclude the possibility that Treg cells contribute to the selective inhibition CD8 T cell priming to shed placental mOVA; however, to be consistent with the data presented here, these cells would have to have a FOXP3^+^ CD25^−^ phenotype and would have to act in a highly localized way that does not inhibit concurrent CD8 T cell responses to the same antigens presented by nearby APCs.

The exact nature of the *cis*-acting pathways that enforces the non-immunogenicity of shed mOVA is currently unclear. Our experiments with CCR7-deficient mice indicate that the APC subset that presents mOVA to CD8 T cells is distinct from both the APC subset that presents intravenously injected sOVA to CD8 T cells, as well as the APC subset that presents mOVA to CD4 T cells. These observations provide an explanation for why sOVA is able to prime OVA-specific CD8 T cells even during Act-mOVA-sired pregnancies, and suggest that the role of the *cis*-acting pathways is to sequester mOVA from immunogenic APCs with cross-presenting capability such as langerin^+^ CD8α^+^ DCs, which are thought to be the main APCs in the spleen that mediate cross-priming to sOVA [35]. It is also possible that mOVA-associated factors specifically modulate the function of ingesting APCs. Importantly, pathways induced by maternal factors might be among those that render placental mOVA non-immunogenic. For example, we previously showed that released mOVA becomes associated with complement components [19], which have known affects on both DC function and antigen disposition in vivo [36], [37].

Although exposure to shed mOVA induced a certain degree of clonal deletion, this process was surprisingly inefficient and allowed for the survival of CD8 T cells that remained competent for activation. Interestingly, this result might also be explained by mOVA sequestration from langerin^+^ CD8α^+^ DCs, since these DCs are also thought be a key APC subset that mediates CD8 T cell tolerance under non-inflammatory conditions [38]. On the other hand, we also found that the adoptive transfer of high numbers of OT-I cells (5×10^5^) occasionally allowed for “break-though” priming to shed mOVA in adjuvant-injected mice that was not observed in mice bearing unmanipulated T cell repertoires. In the context of the “autopilot” model of CD8 T cell priming, which posits that a brief period of antigenic stimulation is largely sufficient to induce expansion and effector differentiation [39], this result suggests that the limits in CD8 T cell expansion to mOVA requires ongoing, repeated T cell interactions with mOVA-presenting APCs. Cell expansion and priming may thus only occur when large numbers of cells overwhelm the ability of mOVA-ingesting APCs from holding their proliferation in check. Indeed, break-through priming explains our previous result that pregnant females bearing Act-mOVA^+^ concepti and given both adjuvants and 1×10^6^ OT-I cells occasionally induced high OVA-specific CTL activity, whereas adjuvant injection induced no OVA-specific priming in the Act-mOVA-mated females analyzed here that bore endogenous T cell repertoires [3]. Break-through priming might also partly explain why transferred OT-I cells show ∼5-fold-increased expansion and elevated IFNγ expression in Act-mOVA-mated pregnant mice with reduced numbers of Treg cells [9], although these effects are also consistent with the more generic role for Treg cells in constraining the magnitude of CD8 T cell priming discussed above.

It is interesting to consider how our results here relate to our prior work on the properties of shed placental mOVA retained by follicular dendritic cells (FDCs), the stromal cell constituents of secondary lymphoid organs that retain complement fixed material for extended time periods [19]. In this study, we showed that FDC-associated mOVA depots are perpetually sampled by LN-resident DCs for several weeks into the postpartum period. Similar to our results here, the presentation of this acquired antigen was highly non-immunogenic and failed to prime transferred OT-I T cells despite adjuvant injection and Treg cell depletion. However, we also found that the acquisition and presentation of FDC-bound OVA:anti-OVA antigen:antibody immune complexes was similarly non-immunogenic, which suggested that it was an antigen's association with an FDC depot *per se* that rendered it non-immunogenic rather than a property of the antigen. On the other hand, we have also found that mOVA does not require binding to FDCs in order to be presented to CD8 T cells during pregnancy (since OT-I T cells transferred during pregnancy still robustly proliferate in Act-mOVA-mated B cell- and FDC-deficient µMT mice) [19], nor to be rendered non-immunogenic (since adjuvant injection into these pregnant mice does not induce CD8 T cell priming to OVA; data not shown, *n* = 3 mice). Recent results demonstrating the constant recycling of FDC plasma membrane-associated immune complexes with non-degradative endosomes [40] provides a possible resolution to this apparent contradiction, as these results raise the possibility that admixing of the regulatory components of non-immunogenic material such as shed mOVA (or similar endogenously generated material) with antigen:antibody immune complexes might render the latter non-immunogenic over time.

Importantly, it is unclear whether the pathways that render shed placental antigen non-immunogenic for CD8 T cells are also relevant to CD4 T cells. In a recent study, the inoculation of pregnant mice with *L. monocytogenes*, which induces systemic inflammation, failed to increase the low fold-expansion of endogenous CD4 T cells specific for an MHC class II-restricted surrogate placental antigen [5]. In this case the surrogate antigen was a mimotope (2W1S) derived from the MHC class II I-E^d^ α-chain and incorporated into the Act-mOVA construct [41]. When the pathogen was engineered to express 2W1S, CD4 T cell expansion reached the same ceiling seen in infected virgins. These results parallel our observations with CD8 T cells, and suggest that *cis*-acting pathways also help enforce the non-immunogenicity of shed placental antigens for CD4 T cells. On the other hand, infection with 2W1S-expressing *L. monocytogenes* induced no IFNγ expression among 2W1S-specific CD4 T cells in postpartum females that had delivered 2W1S^+^ pups [5]. This result was attributed to Treg cells and contrasts with our observation that OVA-specific CD8 T cells remain competent for IFNγ expression in Act-mOVA-mated pregnant females. It is currently unclear whether this discrepancy reflects intrinsic differences between CD4 and CD8 T cells, or distinct pathways of pregnancy-induced immune regulation.

Provocatively, the select non-immunogenic rendering of shed placental material provides a potential way to reconcile the competing demands of pregnancy and host defense. On the one hand it minimizes the possibility that infection-induced APC activation will inadvertently stimulate an effector CD8 T cell response to placental antigens. Even though placenta-specific effector CD8 T cells might be unable to directly attack the conceptus [42], it is likely that their systemic production of inflammatory cytokines would be detrimental to pregnancy success. On the other hand, the specific “tagging” of placental antigens for select non-immunogenicity means that fetomaternal tolerance, at least with respect to the CD8 T cell compartment, does not have to rely upon systemic antigen non-specific immunosuppression. Such suppression, while clearly a quantitative factor in limiting the magnitude of CD8 T cell responses during pregnancy, may thus only modestly diminish immunogenic CD8 T cell responses to pathogens.
